# Adding a “Notch” to Cardiovascular Disease Therapeutics: A MicroRNA-Based Approach

**DOI:** 10.3389/fcell.2021.695114

**Published:** 2021-08-30

**Authors:** Luisa Marracino, Francesca Fortini, Esmaa Bouhamida, Francesca Camponogara, Paolo Severi, Elisa Mazzoni, Simone Patergnani, Emanuele D’Aniello, Roberta Campana, Paolo Pinton, Fernanda Martini, Mauro Tognon, Gianluca Campo, Roberto Ferrari, Francesco Vieceli Dalla Sega, Paola Rizzo

**Affiliations:** ^1^Laboratory for Technologies of Advanced Therapies (LTTA), Department of Translational Medicine, University of Ferrara, Ferrara, Italy; ^2^Maria Cecilia Hospital, GVM Care & Research, Ravenna, Italy; ^3^Department of Medical Sciences, University of Ferrara, Ferrara, Italy; ^4^Cardiovascular Institute, Azienda Ospedaliero-Universitaria di Ferrara, Ferrara, Italy

**Keywords:** Notch, miRNA, atherosclerosis, arrhytmia, heart failure, myocardial ischemia, ischemia-reperfusion injury, calcific aortic valve disease

## Abstract

Dysregulation of the Notch pathway is implicated in the pathophysiology of cardiovascular diseases (CVDs), but, as of today, therapies based on the re-establishing the physiological levels of Notch in the heart and vessels are not available. A possible reason is the context-dependent role of Notch in the cardiovascular system, which would require a finely tuned, cell-specific approach. MicroRNAs (miRNAs) are short functional endogenous, non-coding RNA sequences able to regulate gene expression at post-transcriptional levels influencing most, if not all, biological processes. Dysregulation of miRNAs expression is implicated in the molecular mechanisms underlying many CVDs. Notch is regulated and regulates a large number of miRNAs expressed in the cardiovascular system and, thus, targeting these miRNAs could represent an avenue to be explored to target Notch for CVDs. In this Review, we provide an overview of both established and potential, based on evidence in other pathologies, crosstalks between miRNAs and Notch in cellular processes underlying atherosclerosis, myocardial ischemia, heart failure, calcification of aortic valve, and arrhythmias. We also discuss the potential advantages, as well as the challenges, of using miRNAs for a Notch-based approach for the diagnosis and treatment of the most common CVDs.

## Introduction

**FIGURE 1:**
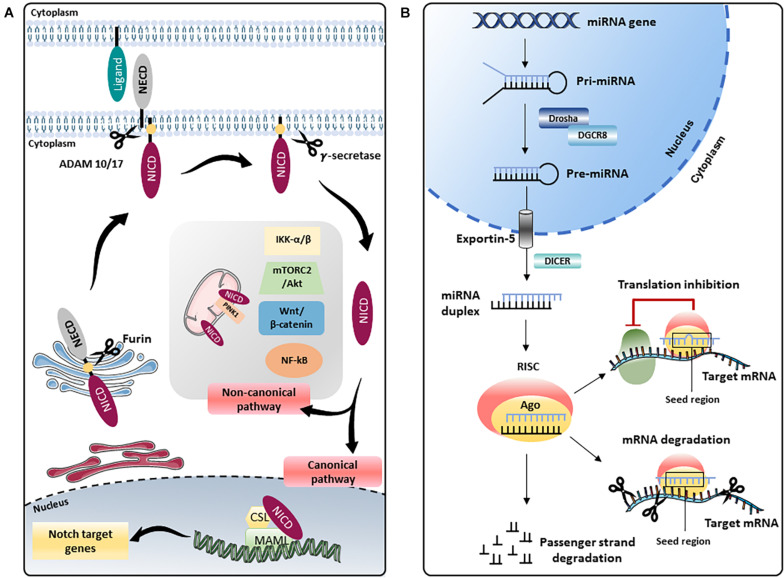
Schematic representation of the Notch pathway and miRNA biogenesis. **(A)** The Notch precursor is cleaved by a furin-like protease in the Golgi apparatus and then transported to the plasma membrane. Notch receptor on the membrane is activated by the binding with ligands present on adjacent cells, which induces a second proteolytic cut mediated by A Disintegrin And Metalloprotease (ADAM) 10 or 17, followed by a third cleavage by the γ-secretase complex that releases the Notch intracellular domain (NICD). In the “canonical” Notch signaling, NICD translocates into the nucleus and, through the interaction with the transcription factor CBF1/Suppressor of Hairless/LAG1 (CSL) and the Mastermind-like (MAML) adaptor proteins, modulates the transcription of several target genes. In the “non-canonical” signaling, Notch modulates Wnt/β-catenin, mammalian target of the rapamycin 2 complex (mTORC2)/Akt, Nuclear Factor kappa B (NF-κB), IkB kinase (IKK)-α/β and phosphatase and tensin homolog (PTEN)-induced kinase (PINK1) on mitochondria, independently from CSL. NECD: Notch extracellular domain. **(B)** In the nucleus, genes coding for miRNAs are transcribed by RNA polymerase II or III into primary miRNAs (pri-miRNAs), which are processed by the complex consisting of RNA-binding protein DGCR8 and type III RNase Drosha, into a stem-loop structure called precursor miRNAs (pre-miRNAs). Afterward, pre-miRNA is transported into the cytoplasm by Exportin-5 complex (Exportin -5^_^ Ran-GTP), where pre-miRNA is processed by another type III RNase enzyme Dicer to form the mature miRNA duplex. The two strands of the duplex are loaded into Argonaute (Ago) protein to form the RNA-induced silencing complex (RISC); one strand (also called guide strand) is selected to form the functional RISC complex, the other strand (passenger strand) is released from Ago and degraded (Medley et al., 2021). For most miRNAs, one strand is preferentially loaded into Ago, forming the functional RISC, while the other is released and degraded. However, for some miRNAs, both strands give rise to functional miRNAs that are loaded into the RISC. Functional miRNAs regulate gene expression at post-transcriptional level mainly by binding their seed region (nucleotides 2–8) to the 3′-untranslated region (UTR) of their target mRNAs. The interaction miRNA/mRNA modulates gene expression by inhibiting translation or inducing degradation of a specific mRNA depending on the complementarity of the sequence.

The Notch pathway, a highly conserved modality of intercellular signaling, is crucial for the development and postnatal homeostasis of the cardiovascular system (MacGrogan et al., 2018). The Notch signaling is activated as a result of the interaction between receptors (Notch 1–4) and ligands (Delta-like 1, 3, 4, and Jagged 1, 2) present on the surface of adjacent cells. The interaction between receptors and ligands triggers a first proteolytic cut which removes the extracellular portion of the receptor generating a membrane intermediate that undergoes a second proteolytic cut mediated by the γ-secretase complex that releases the intracellular active form of Notch (Notch intracellular domain, NICD). Notch intracellular domain translocates into the nucleus and, through the interaction with the transcription factor recombinant binding protein for the immunoglobulin region kJ (RBPJ, also known as CBF1/Suppressor of Hairless/LAG1, CSL) and the Mastermind-like (MAML 1–3) adaptor proteins, modifies the transcriptional landscape of the cell. The best characterized Notch target genes belong to HES (Hairy and Enhancer of Split) and HEY (Hairy / enhancer-of-split related) genes family and are essential modulators of transcription in neuronal, endocrine, and cardiovascular districts (Wiese et al., 2010) [for more details on the Notch pathway the reader is referred to Siebel and Lendahl (2017) and Akil et al. (2021)]. The above described modality of action of Notch is commonly defined as “canonical,” however, there is accumulating evidence of the existence of a “non-canonical” Notch pathway which is activated in the absence of “canonical” ligands (Delta-like 1, 3, 4, and Jagged 1, 2), independent of γ-secretase cleavage and leading to the formation of NICD that can interact with nuclear proteins different from RBPJ and with cytoplasmic proteins. Through the “non-canonical signaling,” Notch modulates pathways such as Wnt/β-catenin, mammalian target of the rapamycin 2 complex (mTORC2)/Akt, Nuclear Factor kappa B (NF-κB), IkB kinase (IKK)-α/β and phosphatase and tensin homolog (PTEN)-induced kinase (PINK1) on mitochondria which, after binding NICD, activates mTORC2/Akt pathway, promoting cell survival (Ayaz and Osborne, 2014) (Figure 1A).

Depending on the cellular context, the Notch signaling plays different, even opposite, roles, but generally, its activation provides a pro-survival stimulus to the cell, and thus, dysregulated activation of Notch favors cancer progression by promoting cancer cells survival (Rizzo et al., 2013). The evidence that the Notch pathway is upregulated in cancer has prompted tremendous efforts for the development of therapeutic strategies able to interfere with cancer progression by inhibiting Notch. Several approaches have been used to inhibit Notch *in vitro* and *in vivo*, such as: (a) γ-secretase inhibitors (GSI), small-molecules that prevent the release of the active form of Notch by inhibiting proteolytic activity of γ-secretase complex; (b) inhibitors of ADAM (A Disintegrin And Metalloprotease), proteases involved in Notch receptors and ligands processing; (c) monoclonal antibodies (mAbs) that specifically target Notch receptors and/or ligands; (d) γ-secretase modulators, small-molecules that modify the proteolytic activity of γ-secretase and, potentially, able to selectively inhibit a specific Notch receptor and, (e) synthetic small molecules able to inhibit the Notch transcriptional activity by blocking MAML, such as CB-103 (Cellestia Biotech AG) and Syntana-4 (Anastasis Biotech) [reviewed in Majumder et al. (2021)]. Delivery of both γ-secretase inhibitors and Dll4 mAb by specific binding of solid lipid nanoparticles to death receptors (DR)-5 on triple negative breast cancer cells is being investigated (Kumari et al., 2021). Several clinical trials targeting Notch in cancer patients using γ-secretase inhibitors have been conducted, and more are ongoing (clinicaltrials.gov). These trials have shown tolerable toxicity but also limited response to these compounds. The scarce efficacy of the γ-secretase inhibitors in the clinic is thought to be due, at least in part, to off-target effects and dose of the administration, which must not be toxic but, at the same time, able to completely block the pathway [for a discussion of the challenges and the state of art on Notch inhibition in cancer the reader is referred to Aquila et al. (2019), Fortini et al. (2019), Moore et al. (2020), and Rizzo et al. (2020)]. Clinical trials testing the safety and efficacy in cancer patients of alternative approaches to γ-secretase inhibitors for Notch inhibition are ongoing (Fabbro et al., 2020). Notch activation is also required for the induction of the inflammatory response. Thus, its inhibition by γ-secretase inhibitors or by mAb has shown efficacy in animal models of pathologies with a recognized underlying inflammatory state, such as chronic obstructive pulmonary disease (COPD) (Danahay et al., 2015), arthritis (Park et al., 2015), and metabolic syndrome (Fukuda et al., 2012): no clinical trials testing the efficacy of Notch inhibition in these contexts have been conducted so far.

In the cardiovascular system, Notch activation prevents apoptosis of cardiomyocytes (Nistri et al., 2017) and endothelial cells caused by different types of insults (Fortini et al., 2017). Thus, activation of Notch in the heart (Ferrari and Rizzo, 2014) and endothelium (Rizzo et al., 2013) could represent a new therapeutic approach against diseases, such as coronary artery disease (CAD) and heart failure (HF), which remain the leading cause of death worldwide despite continued progress in term of diagnosis, prognosis, and treatment (Aquila et al., 2019). One of the challenges in inhibiting Notch in cancers has been the need to develop a cell- specific delivery of the Notch inhibitor to prevent the onset of tumors in tissues, like the skin, where Notch acts as a tumor suppressor gene (Aquila et al., 2019). In analogy to cancer, for the treatment of CAD and HF, Notch activators should be specifically targeted to cardiomyocytes and endothelial cells in order to prevent cell transformation in other tissues and/or the induction of a harmful, dysregulated inflammation. These are major challenges to overcome and they could explain why, so far, Notch activation to interfere with the progression of heart disease and atherosclerosis has been attempted only in animal models (Ferrari and Rizzo, 2014). Therefore, new approaches are needed to explore the feasibility and effectiveness of Notch-based diagnostic tests and therapies for human CVDs.

One possible approach to specifically activate Notch in the heart and endothelium could be the use of Notch-regulating microRNAs (miRNAs or miRs). MiRNAs are single stranded, non-coding RNAs of 21–25 nucleotides in length involved in the post-transcriptional regulation of many genes, including Notch (Figure 1B). MiRNAs have specific characteristics that make them very attractive in terms of drug development. Depending on the biological context, a specific miRNA may need to be inhibited (miRNA-inhibitors) or over-expressed (miRNA mimics). MiRNA-inhibitors (anti-miRs or antagomiRs) are synthetic single stranded miRNA complementary to a specific endogenous mature miRNA and abolish its activity. Conversely, miRNA mimics (agomiRs) are synthetic double-stranded RNA molecules with a sequence identical to the endogenous specific miRNA. However, use of “naked” miRNAs is not recommended because: (1) difficulty passing through cell membrane due to their polarity, (2) induction of immune response, (3) short half-life and limited stability in systemic circulation due to their rapid nucleases-mediated degradation or inactivation, (4) difficulty in cell- or tissue-specific delivery of miRNA. To date, the most common approaches that have been used to deliver miRNAs are: (a) viral vectors, such as adenoviruses, lentiviruses and adeno-associated viruses (AAVs); (b) oligonucleotide-based products, such as miRNA mimics and miRNA inhibitors, with chemical modifications that preserve the integrity of RNA in the systemic environment; (c) cell derived membrane vesicles, such as exosomes, microvesicles (MVs) and apoptotic bodies; (d) nanoparticles (NPs), made up of organic and inorganic components and, (e) 3D scaffold-based systems, such as hydrogels. These miRNAs delivery approaches [thoroughly discussed in Lu and Thum (2019)] allow to deliver the miRNA systemically, by intravenous (Wahlquist et al., 2014) or subcutaneous administration (Bernardo et al., 2014), or locally, through intramyocardial injection of hydrogels (Wang L. L. et al., 2017), catheter-based intracoronary application of antagomiR (Hinkel et al., 2020) and, by subcutaneous injection (Huang D. et al., 2020) or inhalation of NPs (Miragoli et al., 2018).

Many miRNAs have been identified with the potential to treat and diagnose CVDs [for details the reader is referred to Nouraee and Mowla (2015) and Lucas et al. (2018)]. The aim of this Review is to provide an overview of the existing knowledge on miRNAs-mediated Notch regulation in atherosclerosis, myocardial ischemia, heart failure, calcific aortic valve disease, and arrhythmias, and to propose investigations in the cardiovascular setting on crosstalks between miRNAs and Notch signaling reported in other pathologies, such as cancer and cerebral stroke. This knowledge could represent a step forward in the achievement of cell-specific targeting of Notch for the treatment of CVDs.

## Notch in Atherosclerosis

Atherosclerosis is a multistep, chronic inflammatory condition of the arteries widely recognized as the major cause of CVDs (Geovanini and Libby, 2018). The first step of atherosclerosis is the loss of endothelium integrity. Endothelial cells (ECs) are highly plastic cells that can become chronically activated in response to different stimuli, such as disturbed blood flow, exposure to pro-inflammatory mediators, and lipids. Under physiological conditions, stable/laminar shear stress protects the endothelium favoring an anti-inflammatory, vasodilatory, antithrombotic, and quiescent, non-proliferative phenotype. Instead, low and disturbed/oscillatory shear stress promotes endothelial dysfunction (Bretón-Romero et al., 2016; Aquila et al., 2019) by increasing the transcription of pro-atherogenic genes, such as genes involved in the uptake of circulating low-density lipoproteins (LDLs) and in production of pro-inflammatory cytokines. Once accumulated in the subendothelial space, LDLs undergo different modifications, including oxidation by reactive oxygen species (ROS). Oxidized LDLs induce the expression of adhesion molecules on the surface of ECs, such as vascular cell adhesion molecule-1 (VCAM-1), intercellular adhesion molecule-1 (ICAM-1), and E-selectin, which act as chemioattractors for circulating monocytes and T lymphocytes (Kattoor et al., 2019). Once in the sub-endothelium, the monocytes differentiate into macrophages, which phagocytize the oxidized LDL, thus becoming foam cells. Foam cells and macrophages produce growth factors and cytokines that amplify the inflammatory response (Shaposhnik et al., 2007) and recruit extracellular matrix (ECM) proteins- producing vascular smooth muscle cells (VSMCs), leading to the formation of fibrous, lipid-loaded plaques (Bennett et al., 2016). Atherosclerotic plaque can become unstable and break off, leading to thrombus formation, and thus, myocardial infarction (Virmani et al., 2005).

The Notch pathway modulates the functions of each cell type involved in atherosclerosis progression (Aquila et al., 2019). Stable/laminar shear stress upregulates Notch1, which is required for transcription of genes that preserve endothelial function (Theodoris et al., 2015; Aquila et al., 2018). In line with this observation, other studies have shown that stable/laminar blood flow favors the induction of Notch1, which promotes maintenance of endothelial barrier function (Polacheck et al., 2017) and upregulates the anti-apoptotic protein Bcl-2, thus protecting ECs against apoptosis (Walshe et al., 2011). Furthermore, *in vitro* and *in vivo* studies have shown that decreased expression of Notch1 in response to circulating lipids and pro-inflammatory cytokines, such as tumor necrosis factor alpha (TNF-α) and interleukin (IL)-1β, leads to recruitment of monocytes and overexpression of inflammatory molecules (Briot et al., 2015). In support for the protective role of Notch1 in the endothelium, studies *in vitro* have shown that the dysregulation of Notch signaling in ECs induced by inflammation, leads to NF-kB activation and induction of ICAM-1, VCAM-1, and apoptosis (Quillard et al., 2009, 2010; Fazio and Ricciardiello, 2016). In addition, we have shown that the protective effect of 17-β-estradiol against TNF-α induced apoptosis requires Notch1 activation (Fortini et al., 2017). However, the studies discussed so far are in contrast with other observations suggesting a pro-atherogenic and pro-inflammatory role of the Notch pathway in the contest of endothelium (Verginelli et al., 2015; Nus et al., 2016; Poulsen et al., 2018). The features of Notch signaling that could explain these opposite roles of Notch in the endothelium are discussed elsewhere (Aquila et al., 2019).

In VSMCs, the Notch signaling is mediated by Notch 1, 2, 3, and the ligand Jagged1 (High et al., 2007; Tang et al., 2010; Baeten and Lilly, 2015; Rizzo et al., 2018). Boucher et al., showed that Jagged-1 mediated activation of Notch2 significantly inhibited proliferation of human VSMCs via cell-cycle arrest and that high levels of Notch2 were localized to the non-proliferative zone of injured arteries (Boucher et al., 2013). Similarly, the Jagged1/Notch3 axis promotes the VSMCs contractile phenotype (Domenga et al., 2004; Doi et al., 2006; Boscolo et al., 2011) and NF-kB-mediated inhibition of Notch3 favors the transition from a contractile to a secretory, pro-inflammatory phenotype (Clement et al., 2007). Consistent with these studies, we demonstrated that in rat aortic VSMCs, cholesterol accumulation favored the reduction of contractile phenotype and the induction of pro-inflammatory markers in association with low levels of Jagged1 and high levels of Dll4 (Aquila et al., 2017). Furthermore, in VSMCs expression of active form of Notch1 and Notch3 promotes cells survival and inhibition of Notch favors apoptosis (Sweeney et al., 2004). Notch1 seems to be also implicated in proliferation and cell survival in the context of vascular injury (Li et al., 2009) and, consistently, Redmond and colleagues showed that perivascular delivery of siRNA for Notch1 inhibited neointimal formation and VSMCs migration and proliferation (Redmond et al., 2014).

In macrophages, the Notch signaling induces a pro-inflammatory phenotype. Treatment of macrophages with pro-inflammatory stimuli such as lipopolysaccharide (LPS), which promotes the M1 pro-inflammatory phenotype, induces the transcription of Dll4, Jagged1, and Notch1 (Monsalve et al., 2006). In addition, as shown by Fung et al., Dll4-dependent activation of Notch signaling in macrophages leads to an increased inflammatory response (Fung et al., 2007). Conversely, Notch inhibition appears to increase the polarization of macrophages toward an anti-inflammatory M2 phenotype (Singla et al., 2017).

### MiRNAs Which Regulate Notch in Atherosclerosis

Endothelial cell-specific miRNA miR-126 is well-known for its modulation of angiogenesis (Wang et al., 2008), but several *in vitro* and *in vivo* studies also suggest a protective role of both strands of miR-126, namely miR-126-3p and miR-126-5p, against atherosclerosis. MiR-126-3p [also known as miRNA-126 (Cerutti et al., 2017)] reduces endothelial cells injury by restoring autophagic flux (Tang and Yang, 2018) and by regulating VCAM-1 levels and thus, limiting leukocytes-EC interactions (Harris et al., 2008). In support of the atheroprotective effect of miR-126-3p, Zernecke et al. showed that systemic delivery of miR-126-3p induces C-X-C motif chemokine ligand 12 (CXCL12), promoting vascular protection (Zernecke et al., 2009). Likewise, Pei and colleagues demonstrated that miR-126-3p counteracts vascular injury through upregulation of C-X-C Motif Chemokine Receptor 4 (CXCR4) and promotion of stemness gene expression in endothelial progenitor cells (EPCs) (Pei et al., 2020). MiR-126-3p is an important regulator of vascular wall remodeling: its delivery by endothelial microparticles inhibits VSMCs proliferation and neointima formation by targeting LDL Receptor Related Protein 6 (LRP6) (Jansen et al., 2017). Under conditions of high shear stress, miR-126-5p prevents apoptosis, by targeting caspase-3, and promotes autophagy, thus maintaining endothelial integrity (Santovito et al., 2020). Furthermore, Hao et al, highlighted its role in preventing atherosclerosis progression through the targeting of mitogen-associated protein kinase (MAPK) signaling pathway in THP-1 cells (Hao and Fan, 2017), and Schober et al. demonstrated that miR-126-5p delays atherosclerotic plaques formation by reducing endothelium damage through suppression of the Notch1 inhibitor, Delta-like 1 homolog (Dlk1), which promotes ECs proliferation. On the contrary, lower expression of miR-126-5p in response to disturbed flow, suppresses ECs proliferation by upregulating Dlk1 (Schober et al., 2014). Of relevance, plasma levels of miR-126 were found significantly downregulated in CAD patients compared with healthy subjects (Wang X. et al., 2017).

Another miRNA showing a protective effect in the endothelium is miR-107. As demonstrated by Gao et al., in a mouse model of coronary arteries atherosclerosis, miR-107-specific inhibition of KRT1 activates Notch1 protecting against inflammation and endoplasmic reticulum stress in vascular endothelial cells (Gao et al., 2019). On the contrary, in response to hyperlipidemia and oxLDL, Notch1 is inhibited by the upregulation of miR-103-3p, which interferes with lncWDR59 interaction with Numb, a protein involved in the downregulation of Notch (Natarelli et al., 2018).

Experiments in bone marrow endothelial cells have shown that miRNA-155 promotes inflammation by targeting κB-Ras1, an inhibitor of the nuclear factor κB (NF-κB) and that Notch1 activation represses miR-155 expression by promoting binding of RBPJ to the miR-155 promoter (Wang et al., 2014). In the context of cerebral ischemia/reperfusion damage, deletion of miR-155 increased the expression of active Notch1 induced endothelial NO synthase (eNOS) expression and NO production (Jiang et al., 2019). In light of these data, the finding of miR-155 downregulation in the serum of patients with coronary artery disease may be interpreted as protective measure to limit vascular damage (Fichtlscherer et al., 2010).

The miR-143/145 cluster plays a major role in regulating VSMCs functions. Under atheroprotective, laminar shear stress, endothelial vesicle-mediated transfer of miR-143/145 promotes a contractile phenotype in VSMCs (Hergenreider et al., 2012). In addition, *in vitro* and *in vivo* studies have shown that overexpression of miR-143/145 inhibited VSMCs proliferation and neointimal formation by targeting angiotensin-converting enzyme (ACE), Krüppel Like Factor 5 (KLF5), and CD40 (Boettger et al., 2009; Cheng et al., 2009; Cordes et al., 2009; Elia et al., 2009; Guo et al., 2016). Jagged1-mediated activation of Notch1 is required by miR-143/145 to maintain a differentiated phenotype in VSMCs (Boucher et al., 2011). Upregulation of miRNA-146a in VSMCs promotes cells proliferation *in vitro* and vascular neointimal hyperplasia *in vivo*, through the silencing of Krüppel-like factor 4, and its downregulation attenuates platelet-derived growth factor (PDGF) mediated induction of VSMCs proliferation (Sun et al., 2011). miR21 was also found to be upregulated in injured arteries, and downregulation of aberrantly overexpressed miR-21-5p [also known as miR-21 (Dai et al., 2020)] is associated with the inhibition of neointima lesion formation in rat carotid artery after angioplasty (Ji et al., 2007). Of interest, miR-146a and miR-21 increase VSMCs proliferation through suppression of the Jagged1/Notch2 pathway (Cao et al., 2015).

In macrophages, miR-181b suppresses Notch1 and blocks vulnerable lesion development by favoring macrophage polarization toward anti-inflammatory phenotype (An et al., 2017). Similarly, in macrophages and monocytes, miR-146a targets tumor necrosis factor receptor–associated factor 6 (TRAF6), leading to inhibition of NF-κB-driven inflammation and atherosclerosis (Li et al., 2015), and promotes macrophage polarization toward an anti-inflammatory phenotype by inhibiting Notch1 (Huang et al., 2016). MiR-133b downregulation inhibits macrophages migration and the formation of vulnerable atherosclerotic plaques by interfering with the Notch signaling, with molecular mechanisms still partially unknown, which include MAML1 (Zheng et al., 2019). Of interest, both miR-133a and miR-133b have been found upregulated in vulnerable atherosclerotic plaques isolated from patients (Cipollone et al., 2011; Maitrias et al., 2015), thus providing the rationale for the targeting of these miRs in atherosclerosis. MiR-148a-3p, downstream of Notch, promotes a pro-inflammatory M1 phenotype through PTEN/AKT-mediated upregulation of NF-κB signaling (Huang et al., 2017).

## Notch in Myocardial Ischemia/Reperfusion Injury

Myocardial ischemia (MI) results from the rupture or erosion of an atherosclerotic plaque with thrombotic occlusion of an epicardial coronary artery. The myocardium perfused by the occluded artery is in jeopardy, and if the coronary artery is not reopened, the myocardium becomes necrotic. Timely restoration of blood flow, namely reperfusion, is the only effective option to limit myocardial necrosis and then infarct size. However, the salvaged myocardium is never 100% of the myocardium alive at the time of reperfusion, and a large body of experimental and clinical evidence supports the notion that reperfusion may induce additional damage to the myocardium. This damage is called reperfusion injury. Numerous processes are involved in reperfusion injury, including changes in pH, generation of ROS, intracellular Ca^2+^ overload, inflammation (Bonora and Pinton, 2019; Soares et al., 2019), mitochondrial dysfunction and mitophagy, the process that removes defective and not functional mitochondria by autophagy (Patergnani and Pinton, 2015; Morciano et al., 2020), leading to the stimulation of multiple programs of cardiomyocytes death, such as apoptosis, necrosis, necroptosis, and autophagy-associated cell death (Konstantinidis et al., 2012; Bonora et al., 2019).

Notch signaling plays a crucial role in regulating cardiac development by controlling the proliferation and differentiation of cardiomyocytes precursors (Campa et al., 2008; Collesi et al., 2008; Kratsios et al., 2010; Gude et al., 2015). Although it has been reported that Notch1 is turned off in adult cardiac cells, several studies have shown that this pathway is re-activated following myocardial ischemia/reperfusion (I/R) injury (Gude et al., 2008; Kratsios et al., 2010; Li et al., 2011). Specifically, activated Notch limits the extent of the ischemic damage: (a) by reducing oxidative stress (Pei et al., 2015); (b) by regulating mitochondrial function (Zhang et al., 2015) and mitophagy (Zhou et al., 2019); (c) by promoting cardiomyocytes survival through stimulation of phosphatidylinositol 3-kinase (PI3K)/Akt pro-survival signaling (Gude et al., 2008; Kratsios et al., 2010), upregulation of the anti-apoptotic proteins Bcl-2 and Bcl-xl and downregulation of the pro-apoptotic protein Bax (Zhou et al., 2013; Yu and Song, 2014). The activation of the Notch signaling by melatonin (Yu et al., 2015b), G1, an agonist of G protein-coupled estrogen receptor (GPER30) (Rocca et al., 2018), and berberine (Yu et al., 2015a) contributes to the reduction of myocardial I/R damage through the activation of HES1 and of PTEN/Akt signaling. The protective effect of Notch in the infarcted heart may depend not only on its role in reducing the I/R damage but also, at later stages after the MI, by promoting angiogenesis and reducing hypertrophic response and fibrosis (Ferrari and Rizzo, 2014).

### MiRNAs Targeting Notch in Myocardial Ischemia/Reperfusion Injury

A large number of miRNAs have been identified that play a critical role in the context of myocardial I/R injury (Weiss et al., 2012). Studies *in vitro* and *in vivo* of I/R have shown upregulation of several miRNAs, whose inhibition can reduce the I/R damage. MiR-34a-5p is upregulated in rat model of myocardial I/R and, miR-34a-5p knockdown attenuates myocardial I/R injury by promoting Notch1-mediated reduction of H9C2 rat cardiomyoblast cells apoptosis and ROS accumulation (Wang Z. et al., 2019). Similarly, miR-155 overexpression is associated with enhanced ROS production and activation of macrophages in a mouse model of ischemic heart (Eisenhardt et al., 2015), whereas reduced expression of miR-155 attenuates myocardial I/R-induced injury by maintaining physiological levels of hypoxia-inducible factor 1-alpha (HIF-1α), a critical cardioprotective factor (Chen J. G. et al., 2019). Since HIF-1α is a well-known Notch activator (Pistollato et al., 2010), it is tempting to speculate that the detrimental effects on the heart of miR-155 overexpression is due to reduced expression of HIF-1α, which, in turn, inhibits Notch signaling. Consistently, miR-155 downregulation limits cerebral I/R injury by activating the Notch signaling (Jiang et al., 2019). MiR-363 is upregulated during *in vitro* ischemia and, its downregulation protects cardiomyocytes against hypoxia-induced apoptosis through the increase of Notch1 activity (Meng et al., 2017). Similarly, in response to H_2_O_2_ and I/R stress, miR-381 expression is upregulated both in cardiomyocytes and in MI mouse model. MiR-381 overexpression enhances H_2_O_2_ and hypoxia-reperfusion (H/R)-induced cardiomyocytes apoptosis and, vice versa, *in vitro* transfection with miR-381 inhibitor or *in vivo* delivery of miR-381 antagomiR significantly reduces cardiomyocytes apoptosis and infarct size, respectively. The detrimental effect of miR-381 seems to be mediated, at least in part, by reducing the Notch signaling through the inhibition of the expression of Jagged1 (Lu et al., 2018). In H9C2 cells, miR-449a expression increases after hypoxia-reperfusion (H/R) injury, and its inhibition protects cells against H/R-induced apoptosis and necrosis by inducing Notch1 signaling (Cheng et al., 2018). Similarly, in a porcine model of I/R-induced damage, miR-92a is upregulated in cardiac ischemic tissue, and its inhibition results in reduced infarct size, increased angiogenesis, decreased inflammation, and cardiac cells death (Hinkel et al., 2013). In this study, the gene target of miR-92a has not been investigated: based on findings in osteosarcoma cells (Liu et al., 2018) and in gastric cancer cells (Shin et al., 2018) showing that miR-92a targets Notch1, it might be interesting to investigate whether this interaction also occurs in the presence of heart damage caused by I/R.

On the other hand, upregulation of specific miRNAs during I/R can mitigate cardiac injury. MiR-146a is upregulated at an early stage of myocardial I/R in mice (Zhang et al., 2019). MiR-146a overexpression protects H9C2 cells and mouse hearts against I/R-induced apoptosis (Wang et al., 2013) and inhibition of miR-146a exacerbates cerebral I/R damage in mice (Chu et al., 2018). After I/R stress, in rats, intramyocardial injection of human mesenchymal stem cells overexpressing miR-146a determines the reduction of fibrosis and induction of Vascular Endothelial Growth Factor (VEGF) expression in the site of injury, which could promote a reparative angiogenesis (Seo et al., 2017). In VSMCs, miR-146a targets Notch2 (Cao et al., 2015) and, in podocytes, the absence of miR-146a upregulates Notch1 (Lee et al., 2017). On the contrary, in melanoma cell lines, miR-146a targets NUMB, a repressor of Notch1 signaling, determining activation of Notch (Forloni et al., 2014). However, to date, it is not known whether miR-146a activates or inhibits Notch1 in cardiomyocytes. After hypoxic stress, miR-210 was found upregulated in non-apoptotic cardiomyocytes and, delivery of miR-210 through injections with non-viral minicircle vector in the ischemic heart of mice improved cardiac function by promoting angiogenesis and inhibiting cardiomyocytes apoptosis (Hu et al., 2010). Similarly, miR-210 overexpressing mice hearts showed a significant increase in blood vessel density in the peri-infarct zone and reduced infarct size following ischemic injury (Arif et al., 2017). It has also been shown that after cerebral ischemia, the upregulation of miR-210 activates the Notch signaling pathway, contributing to angiogenesis (Lou et al., 2012). This suggests that the pro-angiogenic and pro-survival effect of miR-210 in the heart could be due to Notch activation. A recent study has shown that extracellular vesicles (EVs) released by human induced pluripotent stem cell (iPSC)-derived cardiomyocytes (iCMs) exposed to hypoxic conditions are enriched with miR-106a-363 cluster. This EVs-derived mRNA cluster promotes cell cycle re-entry and proliferation of cardiomyocytes by repressing Notch3 to potentially improve cardiac function in the injured myocardium (Jung et al., 2021).

Hypoxic conditions also lead to downregulation of specific miRNAs. In mice, miR-322 levels were found reduced in the heart after myocardial I/R injury and intramyocardial injection of miR-322 mimic significantly reduced cardiac apoptosis and infarct size by inhibiting the F-box and WD repeat domain-containing 7 (FBXWT), a ubiquitin ligase involved in Notch1 degradation (Chen Z. et al., 2019). Reduced levels of miR-30e have been observed in the serum of patients with myocardial I/R injury (Zheng et al., 2018), a reduction that may represent a protective response of the heart to limit the I/R damage since silencing of miR-30e in H9C2 cells during I/R leads to a decrease in oxidative stress and apoptosis by activating the Notch1/HES1/Akt signaling axis (Zheng et al., 2018). Furthermore, Notch is a target of miR-30 in other cell contexts: miR30e downregulates Dll4 leading to abnormal differentiation of small intestine epithelial cells (Shan et al., 2016) and angiogenesis in Zebrafish embryos (Jiang et al., 2013), and its downregulation induces metabolic inflammation by activating Notch (Miranda et al., 2018).

Endothelial cells in the heart are also a target of I/R injury (Singhal et al., 2010). Under hypoxic conditions, miR-24-3p is upregulated in cardiac endothelial cells causing impairment of angiogenesis both in the mice ischemic heart (Meloni et al., 2013) and in developing Zebrafish (Fiedler et al., 2011). Of interest, in a mouse model of limb ischemia, endothelial overexpression of the active form of Notch1 prevents the miR-24-3p-mediated anti-angiogenic effect and, conversely, the inhibition of Notch enhances the anti-angiogenic effect of miR-24-3p, showing that Notch1 is a target of miR-24-3p (Marchetti et al., 2020).

## Notch in Heart Failure

Heart failure (HF) is a complex syndrome caused by pathological ventricles remodeling, which determines inadequate blood perfusion of tissues and organs. HF typically develops as a result of heart injuries, such as MI, pressure and volume overload, myocarditis, and cardiomyopathy. These conditions cause the dysregulation of cellular and molecular signaling cascades leading to cardiomyocytes apoptosis, ultimately causing myocardial hypertrophy and fibrosis, all contributing to ventricular wall thickening and stiffening that ultimately compromise the heart contractile function (Cohn et al., 2000; Ferrari and Rizzo, 2014). Even in the presence of novel therapeutics treatments, the morbidity and mortality due to HF remain high (Rachamin et al., 2021).

Studies *in vitro* and *in vivo* [reviewed in Aquila et al. (2019)] have shown a dysregulation of the Notch pathway in HF, but only a few studies have been carried out in patients to investigate the involvement of Notch in HF. Oie et al. have shown that the Notch components are differentially expressed in myocardium biopsies from patients with HF (Øie et al., 2010). High levels of Notch ligand Dll1, were found in the serum of patients with HF (Norum et al., 2016) and, similarly, high levels of Dll1 and periostin, a non-canonical Notch ligand, were found in serum from patients with dilated cardiomyopathy (Norum et al., 2017). In both these studies, high levels of Dll1 and periostin were associated with the stage of the disease and with adverse outcome. Furthermore, in a clinical study to assess the safety of DII4 blocking mAb to limit tumor angiogenesis, HF was observed in some patients confirming the critical role of Notch dysregulation in the physiopathology of HF (Smith et al., 2014; Chiorean et al., 2015).

### MiRNAs Targeting Notch in Heart Failure

Overexpression of miRNAs has been reported in animal models of HF and shown to be associated with its progression. Overexpression of miR-25 determines a significant loss of contractile function in a mice model of HF. Conversely, inhibition of miR-25 using an antagomiR (Wahlquist et al., 2014) or tough decoy (TuD) RNA inhibitor (Jeong et al., 2018) markedly halted established HF, improving cardiac function and survival, at least in part, by increasing the expression of sarcoplasmic reticulum calcium uptake pump (SERCA2a). Since the overexpression of miRNA-25-3p inhibits Notch1 signaling and TGF-β-induced collagen expression in hepatic stellate cells (Genz et al., 2019), it would be of interest to investigate if the inhibition of Notch signaling determines the detrimental effects of miR-25 overexpression on cardiac function.

In response to cardiac stress and aging, also miR-34a increases (Boon et al., 2013) and targets the critical Ec-coupler, Junctophilin 2 (Jph2) (Hu J. et al., 2019) and Phosphatase 1 nuclear targeting subunit (PUNTS), causing myocardial insufficiency and progressive HF (Boon et al., 2013). In mice with congenital heart disease, Notch1 is a target of miR-34a (Wu et al., 2018), suggesting that the miR-34a-mediated effect on HF development may be due to Notch1 inhibition. MiR-195 levels are upregulated in the myocardium of patients with HF (Zhang X. et al., 2018). MiR-195-5p stimulates cardiac hypertrophy through mitofusin-2 (MFN2) and F-box and WD Repeat Domain Containing 7 (FBXW7) and upregulates angiotensin II (Ang II) (Wang L. et al., 2019). In colorectal cancer cells, miR-195-5p directly negatively regulates Notch2 expression (Lin et al., 2019). MiR-195-mediated regulation of Notch in the heart has not been investigated. The expression levels of miR-199b-5p are upregulated in biopsies of cardiac tissues from patients with HF and, in mouse models of HF, miR-199b-5p inhibition reduces cardiac hypertrophy and fibrosis (da Costa Martins et al., 2010). Duygu et al. suggest that miR-199b-5p may act as a negative regulator of the Notch1 receptor and Jagged1 ligand in post-infarct heart of mice, resulting in pathological cardiac remodeling (Duygu et al., 2017). The miR-212/132 family is upregulated in failing hearts of mice and humans. This family promotes cardiac hypertrophy (Ucar et al., 2012), and miR-212/132 knock-out mice are protected against pressure overload-induced HF. The miR-212/132 family inhibits FoxO3, an anti-hypertrophic and pro-autophagic transcription factor (Ucar et al., 2012) and an activator of Notch signaling (Gopinath et al., 2014). Notch inhibition by miR-212 has also been observed in mice with ischemic stroke, in which miR-212 promotes endothelial progenitor cells proliferation and tube formation through the inhibition of the Notch signaling (Hu and Dong, 2019). Similarly, miR-375 is increased in patients with HF (Akat et al., 2014) and is associated with cardiac hypertrophy and MI in rodent models (Feng et al., 2014; Garikipati et al., 2015). In mice, subcutaneous injection of anti-miR-375 following ischemia reduces infarct size and improves left ventricular function by decreasing inflammation, apoptosis, and stimulating angiogenesis (Garikipati et al., 2017). MiR-375 disrupts the cardiac development of Zebrafish (Zhuang et al., 2020) by targeting Notch2, but the effect on Notch of the heart has not been investigated. Expression of miR-652 also increases in mice following pressure overload and negatively affects heart function. In this model, inhibition of miR-652 improves cardiac function, reducing cardiomyocytes apoptosis and fibrosis, and preserving angiogenesis. These positive effects could be mediated, at least in part, by the upregulation of Jagged1 (Bernardo et al., 2014). MiR-199a is upregulated in several models of HF (Li Z. et al., 2017) but its role in the injured heart is still not completely understood. MiR-199a-3p inhibition through injection of adeno-associated virus (AAV)-anti-miR-199a reduces cardiac remodeling after MI (Yan et al., 2021). In contrast, miR-199a overexpression stimulates cardiomyocytes de-differentiation and proliferation, determining cardiac regeneration in mice (Eulalio et al., 2012) and pig model of MI (Gabisonia et al., 2019). Furthermore, Gabisonia et al. have shown that one month after MI and delivery of miR-199a, treated pigs showed long-term and non-controlled proliferation of poorly differentiated cardiomyocytes, which determines sudden death due to arrhythmia. In this study, the negative effect of miR-199a has been attributed, at least in part to the simultaneous expression of both miR-199a-3p and miR-199a-5p, which could have a detrimental effect on the heart (Gabisonia et al., 2019). MiR-199a-3p induces Notch signaling in cardiospheres, consisting of both primitive cells and committed progenitors of cardiomyocytes, endothelial cells, and smooth muscle cells, resulting in increased cardiospheres growth to a cardiovascular fate (Secco et al., 2018). However, the effects on heart function of the crosstalk between miR-199a and Notch need to be further investigated.

High levels of miR-99a, a Notch-activating miRNA in a cardiac setting (Jing et al., 2017), are protective for the heart. Overexpression of miR-99a attenuates cardiac hypertrophy in transverse aortic constriction (TAC) mice and in isoprenaline (ISO)/angiotensin-II (Ang II)-induced hypertrophic cardiomyocytes through downregulation of hypertrophic mammalian target of rapamycin (mTOR) signaling pathway (Li et al., 2016).

MiRNAs have been found downregulated in HF, and their overexpression has proven to be beneficial for HF. Lack of miR-26a is involved in hypertension-induced myocardial fibrosis, which leads to HF. Specifically, miR-26a knockout mice developed myocardial fibrosis, whereas spontaneous hypertensive rats overexpressing miR-26a showed reduced blood pressure and myocardial fibrosis. Mechanistically, miR-26a prevents angiotensin II-induced fibrogenesis in cardiac fibroblasts by targeting connective tissue growth factor (CTGF) and Smad4 (Zhang et al., 2020). It has been shown that in a lens fibrosis model, miR-26a and miR-26b inhibits endothelial-to-mesenchymal transition (EMT), a key process in cardiac fibrosis, by directly targeting Jagged1 and suppressing Notch signaling (Chen et al., 2017): no data exist on the relationship between miR-26 and Notch in the cardiac fibrosis. Patients with HF show lower levels of circulating miR-30d correlated with an adverse clinical outcome (Melman et al., 2015; Xiao et al., 2017). In the rat and mouse models of ischemic HF, overexpression of miR-30d protects against cardiomyocyte apoptosis, myocardial fibrosis and improves cardiac remodeling, while miR-30d silencing causes opposite effects (Li et al., 2021). Of interest, in pulmonary fibrosis miR-30d overexpression induces the activation of Notch signaling by regulating Jagged1 (Zhao et al., 2018). To date, the interaction between miR-30d and Notch in cardiac remodeling has not been determined.

When considering a miR-based therapy, it should be kept in mind that miRNAs can play different roles in each myocardial cell type. In a mouse model of HF, the silencing of miR-29 family members (miR-29a, b, and c) specifically in cardiomyocytes protects against cardiac hypertrophy and fibrosis, at least in part, by inhibiting Wnt activation (Sassi et al., 2017) and, *in vitro*, miR-29b-3p inhibition induces cardiomyocytes proliferation by activating Notch2 (Yang Q. et al., 2020). Conversely, in cardiac fibroblasts, miR-29b downregulation promotes pro-fibrotic effects (van Rooij et al., 2008) but, no data exist on the possible interaction between miR-29 and Notch in this context. Both strands of miR-21, miR-21-5p and miR-21-3p, are involved in the regulation of cardiac fibrosis and play different roles in cardiac fibroblasts and cardiomyocytes. MiR-21 [also known as miR-21-5p (Dai et al., 2020)] upregulation in cardiac tissue and changes in circulating levels of this miRNA have been associated with right ventricle dysfunction fibrosis and failure (Reddy et al., 2017). In cardiac fibroblasts, miR-21 induces fibrosis in response to myocardial injury (Thum et al., 2008; Ramanujam et al., 2016). In agreement with this study, in a pig model of HF, the intracoronary infusion of antagomiR against miR-21 reduced cardiac fibrosis and hypertrophy and restored cardiac function mainly through the reduction of fibroblast proliferation and macrophage infiltration (Hinkel et al., 2020). In a mouse model of Ang II–induced cardiac damage, cardiac fibroblasts secrete exosomes enriched with miR-21-3p, which induces cardiomyocytes hypertrophy. Additionally, in the same animal model, antagonism of miR-21-3p by injection of rAAV9 via tail vein prevents the development of cardiac hypertrophy (Bang et al., 2014). Conversely, in injured cardiomyocytes, miR-21-3p and miR-21 over expression has an anti-hypertrophic and anti-apoptotic effect, respectively (Sayed et al., 2010; Yan et al., 2015). Additionally, cardiomyocytes and endothelial cells treated with exosomes isolated from explant heart tissue from patients with HF, which are lacking in miR-21 in comparison to exosomes isolated from healthy donors, show reduced ability to promote proliferation and angiogenesis (Qiao et al., 2019). Of interest, in a rat model of MI, miR-21 overexpression promotes cardiac fibroblast-to-myofibroblast transformation and myocardial fibrosis by targeting the Notch ligand Jagged1 and inhibiting the Notch signaling (Zhou et al., 2018). In agreement with this result, previous studies have clearly shown that Notch counteracts cardiac fibrosis by inhibiting myofibroblast differentiation and proliferation (Fan et al., 2011; Nemir et al., 2014; Boopathy et al., 2015; Zhang et al., 2016). To date, the possible role of the interaction between miR-21 and Notch in cardiomyocytes has not been investigated.

During the last decades many studies have investigated the expression profile of miRNA in HF patient’s serum and/or plasma to identify miRNAs that could be used as diagnostic and prognostic markers. Seventy-two differentially expressed miRNAs have been identified in the serum of HF patients in comparison to control groups. Of these, only 5 miRNAs, namely, miR-1228, miR-122, miR-423-5p, miR-142-3p, and exosomal miR-92b-5p, were differentially expressed in more than one study (Peterlin et al., 2020). In agreement with these data, we recently found that miR-423-5p could represent an independent predictor of left ventricle end diastolic volume (D’Alessandra et al., 2020).

## Notch in Calcific Aortic Valve Disease

Calcific aortic valve disease (CAVD) is a degenerative, inflammatory process characterized by alterations such as fibrosis, lipid and calcium accumulation in the valve leaflets sufficiently severe to result in aortic valve stenosis (AVS), causing hemodynamic changes in the heart (Pasipoularides, 2016; Cho et al., 2018). Aortic valve stenosis is currently the most common valvular heart disease, and to date, there are no effective therapies to prevent or slow down the progression of this pathology (Minamino-Muta et al., 2017). Alteration of functions of ECs, SMCs, and valve interstitial cells (VICs), which form the aortic valve, all contribute to its calcification (Rutkovskiy et al., 2017; van der Ven et al., 2017; Vieceli Dalla Sega et al., 2020).

The involvement of the Notch pathway in calcification of the aortic valve is certain since inherited Notch1-inactivating mutations are associated with severe calcification of the aortic valve (Garg et al., 2005). However, the details on Notch dysregulation leading to AVS have not been fully elucidated. Active Notch in ECs is necessary to prevent calcification. In 2012 Hofmann et al. demonstrated that Jagged1 deletion in murine endothelial cells leads to valve calcification (Hofmann et al., 2012) and, later, Theodoris et al. showed that, in human induced pluripotent stem cell (iPSC)-derived ECs heterozygous nonsense mutations in NOTCH1 cause aortic valve calcification by inducing transcription of genes involved in inflammation, osteogenesis and oxidative stress (Theodoris et al., 2015). Activation of Notch1 seems to prevent osteogenic differentiation in aortic VICs (Nigam and Srivastava, 2009; Acharya et al., 2011). On the contrary, it has been reported that activation of Notch1 promotes the calcification process in VICs (Zeng et al., 2013) and interaction with ECs leads to induction of Notch1/3/4 and HEY1 expression in SMCs, promoting their osteogenic transformation (Kostina et al., 2019). Furthermore, in VICs derived from CAVD patients, activation of Notch1 contributes to pro-osteogenic differentiation (Kostina et al., 2018). Further studies are needed to clarify the role of Notch in the contribution of VICs/SMCs to CAVD.

### MiRNAs Targeting Notch in Calcific Aortic Valve Disease

Several studies reported downregulation of miR-126, miR-30b, miR-26a, and miR-195 in the calcific aortic valve. As discussed in the previous paragraphs, miR-126 is mainly expressed in ECs and protects these cells by activating Notch: we can therefore speculate that its downregulation in calcific aortic valve causes endothelial damage leading to CAVD (Wang H. et al., 2017). miRNA-30b and miR-26a mRNA levels were found decreased in VICs of AS patients. Furthermore, i*n vitro* experiments in human VICs showed that overexpression of miR-30b or miR-26a represses mRNA levels of pro-calcification genes such as SMAD1, SMAD3, and Alkaline Phosphatase (ALP) and increases the expression of Jagged2 and SMAD7, suggesting that the Notch ligand Jagged2 may be required for the inhibition of calcification (Nigam et al., 2010). MiR-195 is downregulated and von Willebrand factor (vWF) increased in aortic valves of AS patients. In aortic VICs isolated from patients undergoing aortic valve replacement, the upregulation of miR-195 reduces aortic valve calcification, reducing calcium accumulation and the expression of the pro-calcification genes, osteocalcin, Runx2, and ALP by suppressing vWF and p38-MAPK signaling pathway (Yang L. et al., 2020). On the contrary, Nigam et al. showed that human VICs treated with miR-195 mimic had increased expression of pro-calcification genes such as BMP2 and RUNX2, and also increased expression of Jagged2 and SMAD7 (Nigam et al., 2010). Therefore, the precise role of miR-195 in CAVD needs to be established.

MiR-146a, miR-34a and miR-21 are overexpressed in CAVD. Petrkova and colleagues found increased levels of miR-146a and miR-34a in valvular tissue of patients with AS (Petrkova et al., 2019). MiR-34a promotes the calcification of the aortic valve by inhibiting Notch1, providing evidence supporting a link between Notch1 inhibition in VICs and CAVD (Toshima et al., 2020). As discussed in the previous paragraph, miR-146a increases VSMCs proliferation by suppressing the Jagged1/Notch2 pathway (Cao et al., 2015) suggesting that this miRNA could also promote AS by inhibiting Notch. Villar et al. found that myocardial and plasma levels of miR-21 were significantly higher in AS patients and that miR-21 is required for myocardial fibrosis induction by pressure overload in AS patients (Villar et al., 2013). It was shown that in cancer stroma miR-21 induces the TGF-β1-stimulated transdifferentiation of cultured fibroblasts into myofibroblasts by targeting Programmed Cell Death 4 (PDCD4), a significant negative predictor of collagen I expression. Of interest, Zhou et al. showed that in a rat model of MI, upregulation of miR-21 induced cardiac fibroblast-to-myofibroblast transformation and myocardial fibrosis by targeting Jagged1 and inhibiting the Notch signaling (Zhou et al., 2018). In this case, it remains to be proven whether increasing levels of miR-21 could accelerate CAVD progression by inhibiting the Notch pathway.

## Notch in Arrhythmias

Cardiac arrhythmias, characterized by the dysregulation of the normal rhythm of the heart, are a cause of significant morbidity and mortality (Kim, 2013). Based on the origin of the arrhythmic focus, arrhythmias are commonly classified in ventricular or supraventricular. The most common type of heart arrhythmia is atrial fibrillation (AF), a supra-ventricular tachycardia. Cardiac arrhythmia shows a complex phenotype, which is the result of a variety of cardiac structural remodeling (fibrosis) and electrical remodeling (dysregulation of ion channels expression and function) (Luo et al., 2015). This cardiac remodeling can be caused by multiple factors, such as genetic, lifestyle, and other pathological stress. A genetic etiology has been established for some pathologies, such as Long QT Syndrome (LQTS), Brugada syndrome, and Timothy syndrome. In particular, loss-of-function mutations in SCN5A, a gene encoding a subunit of the cardiac sodium channel (Nav1.5) (Veerman et al., 2015) have been associated with Brugada syndrome (Bezzina et al., 2013). Given the complexity of the pathology, the treatment of cardiac arrhythmias remains a big challenge and the comprehension of how genetics and other concomitant factors might influence disease is of great importance.

There is evidence involving Notch in the pathogenesis of cardiac arrhythmias. In developing mouse myocardium, Notch1 overexpression determines ventricular pre-excitation that mimics Wolff-Parkinson-White syndrome, a condition associated with an increased risk of fatal arrhythmia (Rentschler et al., 2011). In addition, Notch regulates the expression of genes coding for ion channels and transcriptional factors involved in atrial electrophysiology and, as a result, transient Notch1 re-activation predisposes to atrial arrhythmias in mice (Qiao et al., 2017). Furthermore, the inhibition of the Notch signaling with N-N-(3,5-difluorophenacetyl-L-alanyl)-S-phenylglycine-t-butyl ester (DAPT), a γ-secretase inhibitor, attenuates sympathetic hyper-innervation, which is implicated in the etiology of arrhythmia (Yin et al., 2016). Strong evidence in support of the role of Notch in cardiac arrhythmias comes from a study showing an association between single-nucleotide polymorphism (SNP) of the Notch target gene HEY2 (rs9388451) and the Brugada syndrome (Bezzina et al., 2013). As demonstrated in *Hey2* (+/−) heterozygous knockout mice, in the presence of this SNP, Hey2 affects the normal transmural electrophysiological gradient in the ventricle (Veerman et al., 2017). The link between Notch and arrhythmias has been recently confirmed by a study showing that Notch activation within left atrium cardiomyocytes generates a transcriptomic fingerprint resembling AF and distinct cellular electrophysiologic responses (Lipovsky et al., 2020).

### MiRNAs Targeting Notch in Arrhythmias

MiR-1, a muscle-specific miRNA, plays a crucial role in cardiogenesis and prevention of cardiac hypertrophy. However, under ischemic conditions, the ectopic expression of miR-1 has a pro-arrhythmic effect. Specifically, miR-1 levels are elevated in individuals with CAD and, in the rat model of MI, miR-1 over expression, inhibiting the inward rectifier K+ channel (Kir2.1) and connexin 43 (a cardiac gap junction protein), causes a prolonged action potential duration and slows conduction with consequent arrhythmogenesis. Conversely, reducing miR-1 by an antisense inhibitor in infarcted rat hearts relieves arrhythmogenesis (Yang et al., 2007). These results have also been confirmed in the atrial tissue of patients with persistent AF in which miR-1 expression was inversely correlated with Kir2.1 mRNA and protein levels (Girmatsion et al., 2009). It has also been reported that miR-1 promotes cardiac arrhythmia by altering calcium signaling in rat ventricular myocytes (Terentyev et al., 2009) and dysregulates intracellular trafficking-related genes and pathways (Su et al., 2017).

Crosstalk between miR-1 and Notch have been observed in Drosophila cardiac progenitor cells and mouse embryonic stem cells, in which miR-1 downregulates the Notch ligand Dll1 (Kwon et al., 2005; Ivey et al., 2008), whose intracellular form is an inhibitor of Notch1 (Jung et al., 2011). Additionally, in H9C2 cells, miR-1 targets Notch3 (Xu et al., 2020), but the role of Notch3 in cardiac arrhythmia remains to be investigated. Thus, the reduction of arrhythmogenesis by anti-miR-1 could also be due to the inhibition of Notch. A recent study has shown that increased production of ROS, which occurs during cardiac injury, leads to the oxidation of guanine (8-oxoguanine, o^8^G) at positions 2, 3 and 7 in the seed region of miR-1, resulting in a change in mRNAs target. Specifically, miR-1 with o^8^G in position 7 (7o^8^G-miR-1) acquires the ability to downregulate anti-hypertrophic molecules, such as GATA4, glycogen synthase kinase 3β (GSK3β), and the sarco-endoplasmic reticulum Ca^2+^ ATPase (SERCA2) (Seok et al., 2020), an important regulator of excitation–contraction coupling in cardiomyocytes. GATA4 (Walker et al., 2014) and SERCA2 (Roti et al., 2013) inhibition impairs the Notch activation in intestinal epithelial cells and in human leukemia cells, respectively. Conversely, GSK3β suppression enhances Notch1 activity (Zheng and Conner, 2018). This evidence suggests that the oxidative modification of miR-1 differently modulates the Notch pathway. In conclusion, the effects of miR-1 on Notch signaling in cardiac cells may depend on the cellular context, and thus, more data are needed to unveil the role of Notch in miR-1 arrhythmogenic potential.

Upregulation of miR-208a and miR-34a has been reported in animal models and in patients with AF (Callis et al., 2009; Zhu et al., 2018). Studies in transgenic mice have shown that miR-208a determines pro-arrhythmogenic cardiac remodeling by targeting GJA5, encoding the cardiac gap junction protein connexin-40 (Callis et al., 2009; Li S. et al., 2017). MiR-34a plays an important role in the development of AF, regulating Ankyrin B expression, an adaptor protein associated with arrhythmia (Zhu et al., 2018). For miR-208a, both inhibition and induction of Notch1 signaling have been observed in cardiac cells treated with ketamine, a cardiotoxic agent (Yuan et al., 2019), or subjected to H/R, respectively (Zhang S. et al., 2018). MiR-34a downregulates Notch1 in mice with congenital heart disease (Wu et al., 2018), possibly leading to atrial tachyarrhythmia (Hernández-Madrid et al., 2018). Further studies are needed to establish the role of Notch in the arrhythmias associated with miR-208a and miR-34a. MiR-26 is downregulated in atrial samples from animal models and patients with AF, and this downregulation determines an upregulation of Kir2.1 protein, which may promote AF (Luo et al., 2013). miR-30 is downregulated in dogs with AF and, its overexpression reduces connective tissue growth factor (CTFG) expression, reducing fibrosis and inflammation in the left atrium (Li et al., 2012). MiR-26 inhibits Notch in lens (Chen et al., 2017). In obesity, miR-30 downregulation induces Notch1 signaling and inflammation (Miranda et al., 2018). It remains to be established whether loss of these miRNAs in the heart contributes to AF by the activation of Notch.

## Conclusion

Dysregulation of the Notch pathway underlies atherosclerosis, heart failure, aortic valve stenosis, and arrhythmias. Notch is crucial to promote the survival of cardiomyocytes and endothelial cells and maintain the contractile phenotype of VSMCs. On the other hand, high Notch levels in cardiomyocytes are linked to arrhythmias and promote the inflammatory phenotype in macrophages. Based on these premises, any attempt to target Notch for CVDs will have to be highly cell- specific and able to fine tune the level of activity of this signaling pathway. Due to their specific characteristics, miRNAs could represent a tool able to achieve both aims. In this Review, we focused on crosstalk between Notch and miRNAs dysregulated in CVDs that could be exploited to develop novel therapeutic approaches.

**FIGURE 2:**
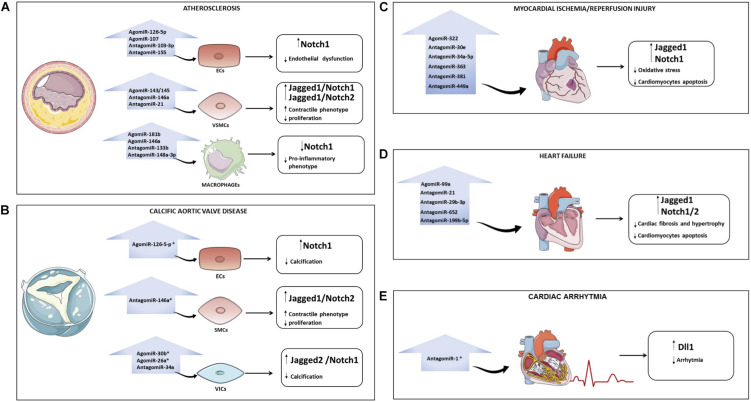
Overview of miRNAs that regulate the Notch pathway in cardiovascular diseases (CVDs). Possible strategies to modulate Notch with miRNAs in CVDs: atherosclerosis **(A)**, calcification of aortic valve **(B)**, myocardial ischemia/reperfusion **(C)**, heart failure **(D)**, and arrhythmia **(E)**. For each pathology, the microRNAs that could be overexpressed (through the use of AgomiR) or downregulated (through the use of AntagomiR) to interfere with disease progression are indicated. The asterisk indicates that the role of miRNAs in specific CVD is still poorly investigated. ECs, endothelial cells; VSMCs, vascular smooth muscle cells; VICs, valve interstitial cells; CAVD, calcific aortic valve disease.

In the context of atherosclerosis, the use of cell-specific Notch- regulating miRNAs could help (i) to reduce endothelium damage (e. g., agomiR-126-5p, agomiR-107, antagomiR-103-3p, and antagomiR-155) and preserve the contractile/differentiated phenotype in VSMCs (e. g. agomiR-143/145, antagomiR-146a and antagomiR-21) by maintaining high levels of active Notch; (ii) to inhibit the release of inflammatory cytokines by macrophages (e. g. agomiR-181b, agomiR-146a, antagomiR-133b, and antagomiR-148a-3p) by inhibiting Notch1 (Figure 2A). In CAVD, restoring miR-126, miR-30b and miR-26a levels or using antagomiR to miR-34a and miR-146a might be a strategy to block disease progression (Figure 2B). Similarly, re-establishing the levels of Notch signaling in cardiomyocytes (e. g. agomiR-322 or antagomiR-30e, -34a-5p, -363, -381 and -449a) may reduce the myocardial damage caused by I/R (Figure 2C). In the context of HF, heart function could be preserved by agomiR-99a or antagomiR-21, -29b-3p, -199b-5p, and -652 able to induce the Notch signaling in the heart (Figure 2D). Treatment based on antagomiR-1 could be a novel therapeutic strategy to reduce AF (Figure 2E). A list of miRNAs able to modulate the Notch pathway in CVDs is provided in Table 1.

**TABLE 1:** Crosstalk between miRNAs and Notch pathway in cardiovascular diseases.

MiRNA	Cardiovascular disease	Cellular type	Details on interaction between miRNA and Notch	Role of miRNA	References
miR-126-5p	Atherosclerosis	ECs	↓Dlk1 →↑Notch1	Preserves endothelial integrity	Schober et al., 2014
miR-107		ECs	↓KRT1 →↑Notch1	Reduces inflammation and endoplasmic reticulum stress	Gao et al., 2019
miR-103-3p		ECs	↓lncWDR59 →↓Notch1	Contributes on ECs maladaptation	Natarelli et al., 2018
miR-155		ECs	Notch →↓miR-155	Promotes inflammation	Wang et al., 2014
miR-143/145		VSMCs	Jagged1/Notch1 →↑miR-143/145	Promotes a contractile phenotype	Boucher et al., 2011
miR-146a		VSMCs	↓Jagged1/Notch2	Promotes proliferation	Cao et al., 2015
miR-21		VSMCs	↓Jagged1/Notch2	Promotes proliferation	Cao et al., 2015
miR-181b		Macrophages	↓Notch1	Promotes anti-inflammatory phenotype	An et al., 2017
miR-146a		Macrophages	↓Notch1	Promotes anti-inflammatory phenotype	Huang et al., 2016
miR-133b		Macrophages	↓ MAML1	Induces migration and vulnerable plaque formation	Zheng et al., 2019
miR148a-3p		Macrophages	Notch →↑miR-148a-3p	Promotes proinflammatory M1 phenotype	Huang et al., 2017
miR-30e	MI/R	Rat cardiomyoblasts (H9C2)	↓Notch1	Increases H9C2 apoptosis and oxidative stress	Zheng et al., 2018
miR-34a-5p		H9C2	↓Notch1	Increases I/R-induced H9C2 apoptosis and ROS accumulation	Wang Z. et al., 2019
miR-322		Mouse cardiomyocytes	↓FBXW7 →↑Notch1	Reduces cardiomyocytes apoptosis and infarct size	Chen Z. et al., 2019
miR-363		H9C2	↓Notch1	Increases hypoxia-induced H9C2 apoptosis	Meng et al., 2017
miR-381		Mouse neonatal cardiomyocytes	↓Jagged1	Increases H_2_O_2_ and hypoxia-induced cardiomyocytes apoptosis	Lu et al., 2018
miR-449a		H9C2	↓Notch1	Increases I/R-induced H9C2 apoptosis	Cheng et al., 2018
miR-21	HF	Rat cardiac fibroblasts	↓Jagged1	Promotes cardiac fibroblast-to-myofibroblast transformation	Zhou et al., 2018
miR-29b-3p		Mouse atrial cardiomyocytes (HL-1)	↓Notch2	Reduces cardiomyocytes proliferation	Yang Q. et al., 2020
miR-99a		H9C2	↑Jagged1/Notch1/Notch2	Reduces H9C2 apoptosis and oxidative stress	Jing et al., 2017
miR-199b-5p		Mouse cardiomyocytes	↓Jagged1/Notch1	Promotes pathological cardiac remodeling	Duygu et al., 2017
miR-652		Mouse cardiomyocytes	↓Jagged1	Promotes pathological cardiac remodeling	Bernardo et al., 2014
miR-126-5p*	CAVD	ECs	↓Dlk1 →↑Notch1	Preserves endothelial integrity	Schober et al., 2014
miR-26a*		VICs	↑Jagged2/SMAD7	Represses calcification related genes	Nigam et al., 2010
miR-30b*		VICs	↑Jagged2/SMAD7	Represses calcification related genes	Nigam et al., 2010
miR-146a*		VSMCs	↓Jagged1/Notch2	Promotes proliferation	Cao et al., 2015
miR-34a		VICs	↓Notch1	Promotes osteogenesis	Toshima et al., 2020
miR-1*	Arrhytmia	Drosophila cardiac progenitor cells	↓Dll1	Promotes cardiac arrhytmia	Kwon et al., 2005

* The role of miRNA in cardiovascular disease is still poorly investigated.

*CAVD, calcific aortic valve disease; ECs, endothelial cells; EPCs, endothelial progenitor cells; HF, heart failure; MI/R, myocardial ischemia/reperfusion; ROS, reactive oxygen species; VICs, valve interstitial cells; VSMCs, vascular smooth muscle cells.*

MiRNAs have been acquiring growing relevance as diagnostic, prognostic markers, and possible drugs in several pathologies (Nouraee and Mowla, 2015; Lucas et al., 2018; Hanna et al., 2019). Among the various miRNAs delivery systems, both viral and non-viral, nanoparticles (NPs) allow the delivery of the miRNA to a specific district, avoiding off-target effects and the induction of immune response. Specifically, NPs can be loaded with antibodies, cell-specific ligands, and aptamers that can recognize a specific cell. To date, NPs targeting macrophages (Hu G. et al., 2019), tumor endothelial cells (Sakurai et al., 2019), cardiac cells (Fan et al., 2020) and VSMCs (Kona et al., 2012) have been developed. When attempting to target Notch for CVDs, this ability of miRNA-loaded NPs to target a specific cell is crucial since, as discussed: (1) the role of Notch signaling is dependent on the cell context and (2) specific miRNAs (e.g., miR-21 and miR-29 in cardiomyocytes and cardiac fibroblasts) can differently affect each myocardial cell type.

MiRNA-based drug therapies are entering into clinical practice. To date, there are several phase I and II clinical trials investigating new miRNA drug candidates (clinicaltrials.gov) (Huang C. K. et al., 2020), including a phase 1b (NCT04045405) investigating the safety and tolerability of a miR-132 inhibitor in patients with stable HF (Täubel et al., 2021). Other clinical trials investigating the efficacy of miRNA for skin disorders could provide crucial information for miRNA-based therapies for CVDs. A phase I clinical trial (NCT03603431) has investigated the effect of intradermal injection of MRG110 (miR-92a inhibitor) on angiogenesis and wound healing in healthy volunteers with small skin wounds. The results of this trial could generate data providing the rationale preliminary data to investigate the efficacy of miR-92a inhibitor in promoting angiogenesis in patients with HF, ischemic cardiomyopathy, and peripheral artery disease. A phase II study (NCT03601052) has investigated the effect of MRG-201 (miR-29 mimic) in preventing or reducing keloid formation in subjects with a history of keloid scar. MiR-29 decreases the expression of collagen and other proteins involved in scar formation (Wang et al., 2012); therefore, this miRNA might show efficacy in the context of extracellular matrix remodeling and myocardial fibrosis occurring in HF. A phase II clinical trial is ongoing to investigate the effect of antimiR-21 in patients with Alport nephropathy and kidney fibrosis (NCT02855268). Of interest, antimiR-21 reduces remodeling and preserves cardiac dysfunction caused by I/R in a large animal model of chronic HF (Hinkel et al., 2020).

Even though the conduction of these early trials justifies a certain enthusiasm, it is clear that further research is required to increase the effectiveness of miRNA-based therapeutics. Among the challenges to be overcome, there is the optimal approach to scale-up production of NPs and the need to obtain more data on clinical safety and long-term stability of loaded miRNAs (van der Ven et al., 2017; Flores et al., 2019).

The use of miRNAs for targeting Notch for CVDs could be even more challenging, given the different roles of this pathway in each cell of the cardiovascular system. Therefore, before attempting the targeting of Notch by miRNA, we will need to identify the most effective miRNA, or combination of miRNAs, to repristinate physiological levels of Notch in the cell type involved in a specific CVD. The knowledge acquired during these last thirty years for the targeting of Notch cancer could be useful in this context. Recently, in an *in vitro* model of triple negative breast cancer, poly (lactic-co-glycolic acid) (PLGA) NPs loaded with miR-34a mimics and Notch1 antibodies are able to stop tumor growth by inhibiting Notch1 signaling and by miR-34a-mediated activation of tumor suppressive genes (Valcourt and Day, 2020). Similarly, targeting the Notch signaling with a combination of miRNAs and other available approaches (Majumder et al., 2021), could successfully achieve the fine tuning of this pathway required to treat CVDs.

## Author Contributions

LM and FF contributed with literature search, manuscript writing, and the figures and table design. EB, FC, and EM contributed to the literature search and writing. PS contributed with the figure and table design. SP, ED’A, RC, PP, FM, MT, GC, and RF contributed with critical reading and intellectual input. FV and PR contributed to the idealization, literature search, and writing. All authors approved the submitted version of the manuscript and agreed to be accountable for the content of the work.

## Conflict of Interest

The authors declare that the research was conducted in the absence of any commercial or financial relationships that could be construed as a potential conflict of interest.

## Publisher’s Note

All claims expressed in this article are solely those of the authors and do not necessarily represent those of their affiliated organizations, or those of the publisher, the editors and the reviewers. Any product that may be evaluated in this article, or claim that may be made by its manufacturer, is not guaranteed or endorsed by the publisher.
